# 3-O-ethyl-l-ascorbic acid: Characterisation and investigation of single solvent systems for delivery to the skin

**DOI:** 10.1016/j.ijpx.2019.100025

**Published:** 2019-07-19

**Authors:** Fotis Iliopoulos, Bruno C. Sil, David J. Moore, Robert A. Lucas, Majella E. Lane

**Affiliations:** a UCL School of Pharmacy, 29-39 Brunswick Square, London WC1N 1AX, UK; b London Metropolitan University, 166-220 Holloway Road, London N7 8DB, UK; c GlaxoSmithKline Consumer Healthcare, St George's Avenue, Weybridge, Surrey KT13 0DE, UK

**Keywords:** *In vitro*, Porcine skin, Permeation, Finite dose, 3-O-ethyl ascorbic acid

## Abstract

l-ascorbic acid (AA), commonly known as vitamin C, has been widely used in topical formulations for many years as an antioxidant and anti-aging ingredient. However, the physicochemical properties of AA are not optimal for skin uptake and the molecule is also unstable, readily undergoing oxidation on exposure to air. The compound 3-o-ethyl-l-ascorbic acid (EA) has been developed as a stable vitamin C derivative and has been used in topical products. The aims of this work were to conduct a comprehensive characterisation of physicochemical properties of EA as well as to investigate the influence of various neat solvents on EA skin delivery. Nuclear magnetic resonance (NMR), mass spectroscopy, differential scanning calorimetry (DSC) and thermogravimetric analysis (TGA) were used to characterise the molecule. The pK_a_ of the compound and the partition coefficient logP_(o/w)_ were experimentally determined. A new HPLC method for analysis of the molecule was also developed and validated. A number of solvents for topical preparations were selected based on their wide use as excipients in topical formulations, their potential to act as skin penetration enhancers and their favourable safety profiles. The solubility and stability of EA was examined. Skin permeation of the molecule in full thickness porcine skin *in vitro* was investigated using Franz-type diffusion cells. The melting point, log P_(o/w)_ value and pK_a_ value of EA were determined to be 114.39 ± 0.5 °C, −1.07 ± 0.03 and 7.72 ± 0.01 respectively. Skin penetration of EA was evident for the following vehicles 1,2 hexanediol (HEX), glycerol (GLY), propylene glycol (PG), 1,2 pentanediol (1-2P), isopropyl alcohol (IPA), propylene glycol monolaurate (PGML) and propylene glycol monocaprylate (PGMC). Skin uptake but no permeation through the skin was observed for Transcutol® (TC) and dipropylene glycol (DiPG), while no penetration was observed for the solvents 1,5 pentanediol (1-5P) and tripropylene glycol (TriPG). The findings of the permeation experiments confirm the potential of simple formulations to deliver EA to the skin. Studies are ongoing to identify complex vehicles for synergistic enhancement of EA skin penetration. To our knowledge this is the first study to conduct a comprehensive characterization of EA and examine its skin uptake and permeation properties in porcine skin.

## Graphical abstract

**Figure:**
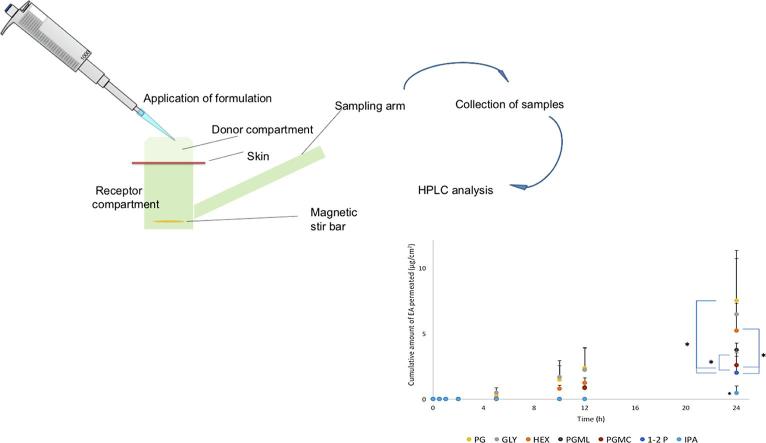

## 1 Introduction

L-ascorbic acid (AA), or vitamin C, is a water-soluble antioxidant that has been used in personal care formulations for many years. A number of studies in cultured cells have been used to explore the mechanism of action of AA *in vitro*. The molecule was shown to protect cells against UV-induced oxidative damage by scavenging free radicals and reactive oxygen species (Colven and Pinnell, 1996, Chan, 1993). AA has also been shown to stimulate collagen synthesis in human dermal fibroblasts, being co-factor for the formation of hydroxyproline and hydroxylysine (Boyera et al., 1998, Kameyama et al., 1996, Pielesz et al., 2017, Chung et al., 1997, Dumas et al., 1996) and it has an anti-tyrosinase effect, inhibiting melanogenesis (Kameyama et al., 1996).

In recent years, the actions of topically applied AA on human skin have been investigated in a limited number of *in vivo* studies. The ability of AA to protect against UV-induced oxidative damage has been examined by Humbert et al. (2003) in a 6-month clinical trial. Specifically, these authors investigated the antiaging effect of topical AA on the sun-exposed skin of the lower neck and forearm of 19 healthy female volunteers. A commercial cream containing 5% AA was applied daily over a period of 6 months and was reported to lead to a statistically significant reduction in clinical signs of photo-ageing when compared with a placebo formulation.

Nusgens et al. (2001) conducted a clinical study with 10 volunteers for 6 months to examine the effects of AA on collagen synthesis. A water-in-oil emulsion containing 5% AA was applied once daily on the dorsal side of the upper forearm, while a control formulation with no AA was applied to the other side. Biopsies were taken from the treated sites and an increase of the mRNA of collagen type I and III as well as the mRNA of three extracellular procollagen processing enzymes was observed for the AA-treated sites compared with the control.

Finally, Espinal-Perez et al. (2004) examined the depigmentation effect of topical 5% AA on 16 female patients’ facial skin with melasma over a 16-week period. The patients applied AA emulsion on one side of their face, and a 4% hydroquinone emulsion on the other side of their face. Hydroquinone is a known bleaching agent for the skin (Jimbow et al., 1974) and the researchers compared the pigmentary changes on the AA treated sites with those on the hydroquinone treated sites. Measurements of melanin index were taken at the beginning and at the end of the study, using a narrow-band reflectance spectrophotometer. AA showed similar lightening effect on the hyper-pigmented areas of the skin as hydroquinone. In a later study, Fitzpatrick and Rostan (2002) compared the clinical changes in 5 patients with moderate or severe dyspigmentation after daily application of a formulation containing 10% AA and 7% tetrahexyldecyl ascorbate for 12 weeks. However, these researchers reported no significant depigmentation on the AA treated sites when compared with the control.

Chemically, AA is a dibasic acidic unsaturated lactone, which consists of an electron rich C_2_ - C_3_ enediol moiety in a five-membered ring. This structure predisposes the molecule to oxidation and instability under either aerobic or anaerobic conditions. More specifically, the delocalization of π electrons in the molecule and the 2,3-enediol moiety conjugated with the C_1_ carbonyl group, make the proton on the C_3_ hydroxyl group significantly more acidic (pK_1_ = 4.25) than the proton on the C_2_ hydroxyl group (pK_2_ = 11.57) (Benedini et al., 2011, Kibbe, 2009). The C_2_ - and C_3_ - OH groups are critical in electron donation and therefore oxidation of AA (Deutsch, 1998, Finholt et al., 1965, Lavrenov and Preobrazhenskaya, 2005).

The rapid degradation of AA poses challenges for topical delivery of the molecule in cosmetic and pharmaceutical products. Moreover, the physicochemical properties of AA such as the melting point (190 – 192 °C) partition coefficient (log P_(o/w)_ = −1.85) and dissociation constant (pK_a_ = 4.25) are not optimal for transport across skin. Attempts have been made to synthesise stable AA derivatives for topical delivery, including salts of l-ascorbyl 2-phosphate or l-ascorbyl 6-palmitate (Austria et al., 1997, Pinnell et al., 2001). However, Austria and co-workers (1997) reported that the derivatives with a C_6_-OH esterified group, like l-ascorbyl 6-palmitate, did not show improved stability in aqueous solutions nor in topical formulations.

3-O-ethyl-l-ascorbic acid (EA) is an l-ascorbic acid derivative with an ethyl group at the third carbon position. This structural modification protects the 3-OH group from ionization, and thus the molecule from oxidation, but also results in changes in the physicochemical properties. With regards to EA safety, to date, only two cases of allergic contact dermatitis have been reported in the literature. The first case was reported in 2014 (Yagami et al.), when a female patient developed an itchy erythematous rash after applying a 2% EA lotion to the face. Patch testing with the ingredients of the formulation indicated that EA was the causative allergen and showed the minimum positive concentration to be 0.05% EA. One year later, a second case of allergic contact dermatitis due to EA were reported. A 51-year female was found to have an allergic reaction to EA after patch testing, with a minimum positive concentration of 1% EA (Numata et al., 2015).

Even though EA is a compound widely used in topical products (Jeon et al., 2016, Silva et al., 2019), surprisingly, its physicochemical properties have not been reported to date. Additionally, the skin permeation profile of EA has not been investigated nor have the effects of different vehicles on skin uptake of EA been examined. The objectives of the present work were therefore (i) to undertake a comprehensive characterisation of EA, (ii) to assess its solubility and stability in a range of solvents commonly used in topical preparations and (iii) to investigate skin delivery of EA from these solvents.

## 2 Materials and methods

### 2.1 Materials

EA (Corum Inc., Taiwan) was a kind gift from Glaxo Smith Kline (GSK, U.K.). Propylene glycol (PG), 1–2 pentanediol (1-2P), 1–5 pentanediol (1-5P), 1–2 hexanediol (HEX), isopropyl alcohol (IPA), isopropyl myristate (IPM) and glycerol (GLY) were supplied by Sigma Aldrich, UK. Transcutol® (TC), Capryol 90® or propylene glycol monocaprylate (PGMC) Type II, Lauroglycol 90® or propylene glycol monolaurate (PGML) Type II and Labrafac® or medium chain triglycerides of caprylic (C_8_) and capric (C_10_) acids were a kind donation from Gattefossé (St. Priest, France). Dipropylene glycol (DiPG), tripropylene glycol (TriPG) and n-octanol (99% pure) were obtained from Acros Organics (UK). High Performance Liquid Chromatography (HPLC) grade water, methanol and *ortho*-phosphoric acid (H_3_PO_4_) were purchased from Fisher Scientific, UK. Phosphate-buffered saline (PBS) tablets were purchased from Oxoid Limited, UK.

### 2.2 Methods

#### 2.2.1 Thermal analysis

The melting point of EA was examined using thermogravimetric analysis (TGA) and differential scanning calorimetry (DSC). TGA was performed using a Discovery TGA (TA Instruments, U.S.A.) system. The active was weighed in an open aluminium pan (TA Instruments, U.S.A.) and then heated inside the Discovery TGA furnace. The starting temperature and the final temperature were set to 25 °C and 500 °C, respectively, while the heating ramp was 10 °C/min. A steady nitrogen flow of 25 ml/min was supplied throughout the analysis in order to create an inert atmosphere around the sample. A DSC Q2000 (TA Instruments, U.S.A.) system was used for the DSC analysis. The active was weighed in a hermetic aluminium pan (TA Instruments, U.S.A.) which was subsequently sealed with a hermetic aluminium lid (TA Instruments, U.S.A.) using a Tzero^TM^ press (TA Instruments, U.S.A.). An empty hermetic aluminium pan (sealed with a hermetic aluminium lid) was used as a reference. Both the sample and reference were heated from 25 °C to 220° C, with a heating ramp of 10° C/min and nitrogen flow of 50 ml/min.

#### 2.2.2 UV, NMR and mass spectra studies

The UV spectra of EA at a concentration of 0.002% w/v in HPLC water, methanol, an acidic aqueous solution and an alkaline aqueous solution were obtained using a Spectronic Bio Mate^TM^ 3 UV/VIS spectrophotometer (Thermo Scientific, U.S.A.). The acidic medium was 0.01 M HCl, pH = 2, and the basic medium was 0.01 M NaOH, pH = 12 (OECD, 1981a). The UV absorption spectrum was acquired between 200 and 300 nm (step = 1 nm) in order to determine the optimum specific wavelength (λ) for analysis of the molecule.

^1^H NMR and ^13^C NMR were collected using a Bruker AM500 spectrometer operating at 500 MHz for proton and 126 MHz for carbon. Chemical shifts (δ_H_ and δ_C_) are quoted as parts per million downfield from 0. The multiplicity of a ^1^H NMR signal is designated by one of the following abbreviations: s = singlet, d = doublet, t = triplet, q = quartet, br = broad and m = multiplet. High-resolution mass spectra were carried out using either a Kratos MS89MS with Kratos DS90 software or a Jeol AX505W with Jeol complement data system. Samples were ionised electronically (EI), with an accelerating voltage of »6kV or by low resolution fast atom bombardment (FAB) in a thioglycerol matrix.

#### 2.2.3 pK_a_ determination

The dissociation constant of EA in water was determined according to OECD guidelines (1981b). Briefly, the UV absorption spectrum was obtained from solutions of fixed concentrations of EA (0.002% w/v) at various pH values from 2 to 12. The absorbance of non-charged and the charged compound was measured with 0.01 N hydrochloric acid (HCl, pH = 2) and 0.01 N sodium hydroxide (NaOH, pH = 12) solutions, respectively. The UV absorption spectra for the dissociated and undissociated forms of EA were found to vary considerably at 275 nm, therefore UV absorption spectra were obtained at several intermediate pH values from 6.80 to 8.61. The various buffered solutions were prepared by adding equal volumes of a stock solution of EA in HPLC grade water to constant volumes of McIlvaine buffer solutions, prepared as described by Elving et al. (1956). The buffered solutions at pH 8.01 and 8.61 were prepared with boric acid and NaOH. The ionic strength was kept constant for all the solutions by adding potassium chloride (KCl) when needed. The pK_a_ was calculated as described by Albert and Serjeant (1984). All pH measurements were taken using a symphony Dual Parameter pH metre (VWR International, U.K.) at room temperature.

#### 2.2.4 HPLC analysis and method validation

The HPLC system consisted of a Hewlett-Packard (U.S.A.) series 1100 quaternary pump, an Agilent Technologies (U.S.A.) series 1100 auto sampler, a Hewlett-Packard (U.S.A.) series 1100 system controller, an Agilent Technologies (U.S.A.) series 1100 degasser and an Agilent Technologies (U.S.A.) series 1100 UV detector. ChemStation® Rev. A.09.03 (Agilent Technologies, U.S.A.) software was used to analyse the data. The HPLC system was equipped with a Luna® column, 250 × 4.60 mm, 5 μm, Phenyl-Hexyl (Phenomenex, U.K.) equipped with a universal HPLC guard column (Phenomenex, U.K.) packed with a SecurityGuard cartridge (Phenomenex, U.K.). The mobile phase consisted of methanol:water [0.08% v/v orthophosphoric acid] (20:80). The UV detector was set to 242 nm, the flow rate to 1 ml/min and the column temperature to 30 °C. The injection volume was 5 μl. The analytical method was validated for linearity, range, precision, accuracy, limit of detection, limit of quantitation, robustness and system suitability according to the International Conference on Harmonization guidelines (ICH, 2005).

A calibration curve ranging from 0.5 to 200 µg/ml was constructed to relate the concentration of the drug in solution to the peak area measurements obtained from the HPLC. A correlation coefficient value (r^2^) >0.99 was obtained, which indicates an acceptable fit to the regression line (Shabir, 2003). The retention time of EA was 7.62 ± 0.07 min. The accuracy, being the closeness of the measured value to the true value, was determined by preparing and injecting three different concentrations of EA and the findings were expressed as mean % recovery. A recovery of 99.51 ± 0.38% was achieved. Precision was assessed by determination of inter-day variability and intra-day repeatability. The % RSD values of intraday and inter-day precision were 0.13% and 1.40%, respectively. The LOD value was 0.10 µg/ml and the LOQ was 0.31 µg/ml. A sample HPLC chromatogram of EA detected in the receptor solution during a permeation experiment is shown in Fig. S1.

#### 2.2.5 Log P_(o/w)_ determination

The log P_(o/w)_ value of EA was determined experimentally following the shake flask method according to the OECD guidelines (1995). Briefly, before the study, n-octanol and HPLC grade water were mutually saturated at the temperature of the experiment, 24 ± 1 °C. A stock solution of EA at 0.0049 M (1 mg/ml) in water pre-saturated with n-octanol was prepared. In Eppendorf® tubes 2 ml, a stock solution of drug and the pre-saturated solvent were taken in the following ratios: 1: 1, 1: 0.5 and 0.5: 1. The tubes were then rotated through 180 °C about their transverse axes (approximately 100-times for 5 min). Subsequently, the tubes were centrifuged at 24 °C at 13,000 rpm for 20 min so that the two phases separated from each other. Samples were first taken from the n-octanol phase and diluted with ethanol. Subsequently, samples from the aqueous phase were collected, diluted and analysed by HPLC. All samples were prepared in duplicate.

#### 2.2.6 Solubility parameter, solubility and stability studies

The van Krevelen and Hoftyzer solubility parameters (δ) of the drug and solvents were calculated using Molecular Modeling Pro® software (Version 6.3.3, ChemSW, CA, USA). For solubility determinations, excess EA was added to each solvent in Eppendorf® tubes in triplicate. The tubes were capped and sealed with Parafilm® (Bemis Inc., U.S.A.) and placed in a shaking incubator for 48 h at 32 ± 1 °C. Tubes were checked periodically to ensure an excess of the active remained in each tube. After 48 h each tube containing saturated mixtures was centrifuged for 15 min at 10,000 rpm and 32 °C. The supernatant liquid was diluted with methanol in order to lie within the analytical range of the HPLC method.

Stability of EA in the solvents was investigated for 120 h (5 d) at 32 ± 1 °C. Solutions of a known concentration of EA were prepared in triplicate and placed in an Eppendorf ® tube. The sample was sealed with Parafilm® (Bemis Inc., U.S.A.) and placed in a shaking incubator, as for solubility studies. Samples were collected at 0, 24, 48, 72, 96 and 120 h. All samples were subsequently diluted and analysed by HPLC.

#### 2.2.7 Finite dose permeation and mass balance studies

EA solutions (2% w/w) were prepared by placing a suitable amount of active ingredient in an Eppendorf® tube containing a Teflon® coated magnetic stir bar. The permeation studies were conducted using vertical glass Franz-type diffusion cells and full thickness porcine ear skin according to procedures reported previously (Haque et al., 2017, OECD, 2004). The diameter of both the donor and the receptor compartments of each Franz-cell were measured using an electronic digital micrometre (Fisher Scientific, U.K.). The experiments were conducted in a SUB 28 (Grant Instruments, U.K.) temperature-controlled water bath equipped with a Telesystem HP 15 (Variomag®, U.S.A.) submersible magnetic stirrer. Freshly prepared PBS (pH 7.3 ± 0.1) was used as the receptor solution. Once the skin temperature had equilibrated to 32 ± 1 °C, 5 μl of the test solution were applied to the donor compartment which was not occluded. A volume of 200 μl of receptor solution was collected at 0, 15 min, 30 min, 1 h, 2 h, 5 h, 10 h, 12 h and 24 h, and equal volume of fresh temperature equilibrated PBS solution was added to the receptor compartment. All samples were analysed using HPLC. The number of replicate experiments was n ≥ 5.

At the end of the permeation studies, the receptor solution was removed from the Franz cells. A mass balance study was also conducted and validated. The skin surface was washed once with 1 ml of methanol and three times with 1 ml of water:methanol (50:50), followed by swabbing with a cotton bud. The Franz diffusion cells were disassembled, the skin membranes were cut into small pieces with scissors and placed in Eppendorf® tubes with 1 ml of water:methanol (50:50). The skin samples were centrifuged for 1 min at 13,000 rpm at 32 °C and subsequently they were extracted by incubation for 5 h in an orbital shaker at 32 °C. All samples were centrifuged at 13,000 rpm at 32 °C for 20 min and the supernatant solution was analysed using HPLC.

#### 2.2.8 Data analysis

The data was analysed in MS Excel® (Microsoft Corp., USA). Statistical significance was determined using one-way analysis of variance (ANOVA). Multiple comparisons between groups were performed by post hoc Tukey test. A probability of p < 0.05 was considered statistically significant. All results are presented as the mean ± SD.

## 3 Results and discussion

### 3.1 Thermal analysis

**Fig. 1:**
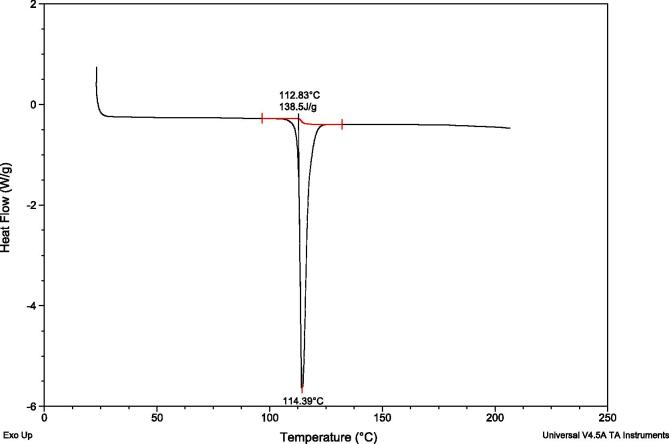
DSC analysis of 3-o-ethyl-l-ascorbic acid.

**Fig. 2:**
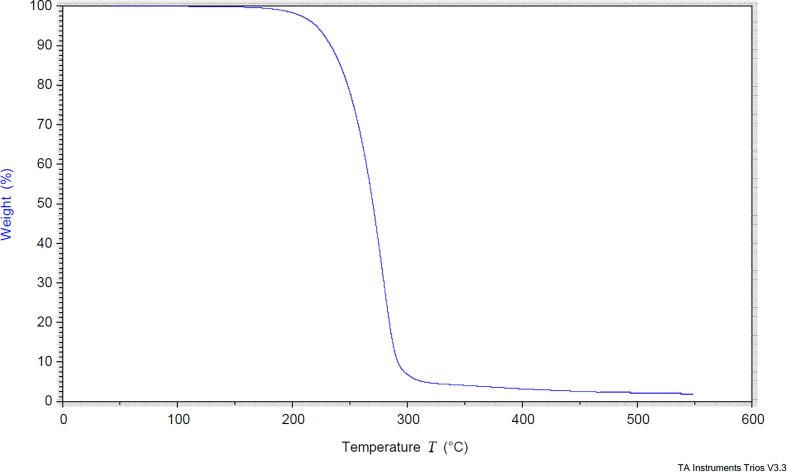
TGA analysis of 3-o-ethyl-l-ascorbic acid.

One single endothermic peak was observed in the DSC curve at 114.39° C, with an extrapolating onset at 112.83° C and enthalpy of fusion 138.5 J/g (Fig. 1). Mass loss in the TGA was observed after 190° C (Fig. 2), a temperature above the melting observed in the DSC, indicating decomposition. This allowed identification of the pure compound, with no impurities or polymorphism evident.

### 3.2 Ultraviolet, NMR and mass spectra

**Fig. 3:**
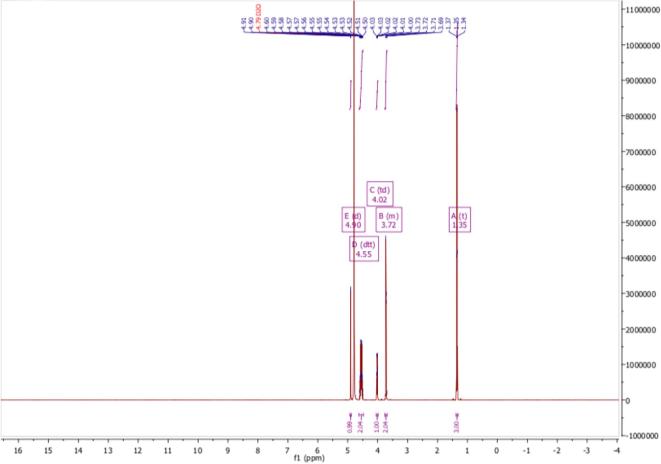
^1^H NMR spectrum of 3-o-ethyl-l-ascorbic acid in D_2_O.

**Fig. 4:**
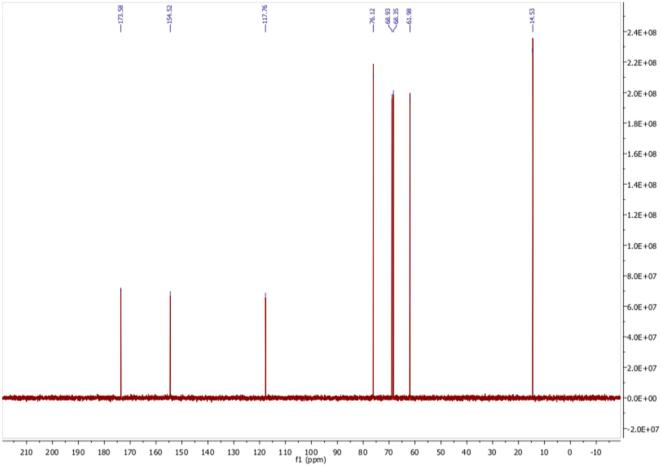
^13^C NMR spectrum of 3-o-ethyl-l-ascorbic acid in D_2_O.

The UV maxima of EA in various solutions are shown in Table 1. The NMR spectra are shown in Fig. 3, Fig. 4.

**Table 1:** UV maxima of EA in various solutions.

	λmax (nm)
Distilled water	242–243
Acidic solution (HCl 0.01 N)	242
Alkaline solution (NaOH 0.01 N)	275
Methanol	241–242

*(±)-(2R)-2-[(1S)-1,2-dihydroxyethyl]-3-ethoxy-4-hydroxy-2H-furan-5-one* White powder; m.p. 114.39 °C; δ H (500 MHz; D_2_O) 1.35 (3H, t, C*H*_3_CH_2_OC[=C(OH)]CH), 3.69 – 3.76 (2H, m, CH_3_C*H_2_*OC[=C(OH)]CH), 4.02 (1H, t, HOCH_2_C*H*(OH)C(H)OC = O), 4.55 (2H, d, HOC*H_2_*CH(OH)C(H)OC = O), 4.90 (1H, d, CH(OH)C(*H*)OC = O); δ H (500 MHz; DMSO‑*d*_6_) 1.27 (3H, t, C*H*_3_CH_2_OC[=C(OH)]CH), 3.37 – 3.47 (2H, m, CH_3_C*H_2_*OC[=C(OH)]CH), 3.60 – 3.68 (1H, m, HOCH_2_C*H*(OH)C(H)OC = O), 4.34 – 4.48 (2H, m, HOC*H*_2_CH(OH)), 4.73 (1H, d, HOCH_2_C(H)O*H*), 4.84 (1H, t, *H*OCH_2_CH(OH)), 4.95 (1H, d, CH(OH)C(*H*)OC = O), 8.69 (1H, s, C = C(O*H*)C = O); δ C (126 MHz; D_2_O) 14.53 (**C**H_3_CH_2_OC), 61.98 (CH_3_**C**H_2_OC), 68.35 (HO**C**H_2_CH(OH)C), 68.93 (HOCH_2_**C**H(OH)C), 76.12**C**(H)OC = O, 117.76 (O = C**C**(OH) = C), 154.52 (HOC **= C**(OCH_2_CH_3_), 173.58 (**C** = O); δ C (126 MHz; DMSO‑*d*_6_) 15.31 (**C**H_3_CH_2_OC), 61.76 (CH_3_**C**H_2_OC), 66.56 (HO**C**H_2_CH(OH)C), 68.49 (HOCH_2_**C**H(OH)C), 74.43**C**(H)OC = O, 118.84 (O = C**C**(OH) = C), 150.55 (HOC **= C**(OCH_2_CH_3_), 170.62 (**C** = O); *m*/*z* 205 (100%, [M + H]^+^): Found [M + H]^+^ 205.0710, C_8_H_12_O_6_ requires 205.0712. Consistent with previous report (MSDS, 2012).

### 3.3 pK_a_ and log P_(o/w)_ determination

The pK_a_ value was calculated as described by Albert and Serjeant (1984), according to the equations pK_a_ = $H+\log\frac{A_{I}-A}{A-A_{M}}$ , where A_I_ is the absorbance of the ionised species and A_M_ the absorbance of the molecular species. EA was found to have a pK_a_ of 7.72 ± 0.01 at room temperature. The log P_(o/w)_ of EA was experimentally found to be −1.07 ± 0.03.

### 3.4 Solubility of EA in candidate solvents

**Fig. 5:**
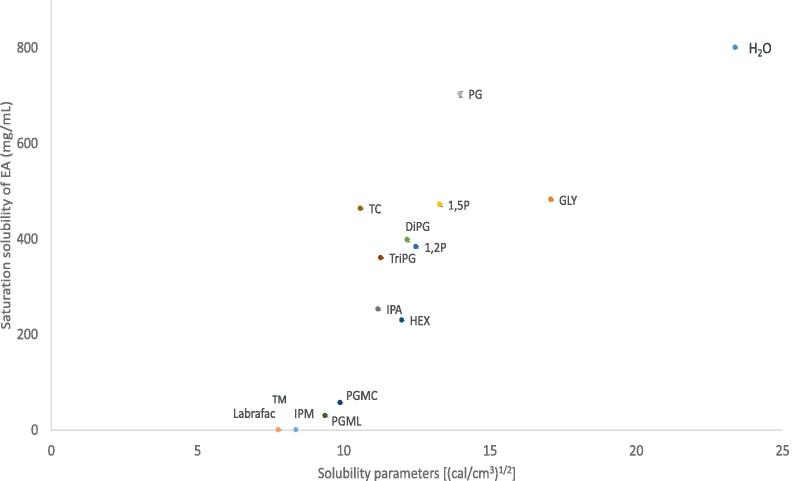
Solubility parameter values of various solvents plotted against saturation solubility of 3-O-ethyl-l-ascorbic acid in vehicles at 32 ± 1 °C, n ≥ 3, mean ± SD.

The saturation solubility values of EA in the solvents studied are shown in Fig. 5. The calculated solubility parameters of vehicles are also shown. The solubility parameter of EA was calculated as 15.4 ((cal/cm^3^)^1/2^). EA solubility ranged from 463.6 ± 1.3 mg/ml to 801.1 ± 1.7 mg/ml for the hydrophilic solvents. Lower solubility was found for the lipophilic solvents Lab, IPM, PGML and PGMC, ranging from 0.6 ± 0.2 mg/ml to 56.6 ± 1.3 mg/ml. Of all the vehicles examined, EA was found to be less stable in TriPG and DiPG after 120 h, with recovery values of 76.64 and 82.76% respectively. In all other solvents, recovery of EA ranged from 90.23% to 101.51%, as shown in Fig. S2.

The solvents were selected based on the hypothesis that solvents with low solubility parameters (δ) could enhance the permeation of the active. This would be expected because of the subsequent low saturation solubility and therefore the high thermodynamic activity of EA in these solvents. Additionally, it has been reported that when the solubility parameter of the vehicle is similar to that of skin, that is 10 (cal/cm^3^)^1/2^ (Liron and Cohen, 1984), then the active will have increased affinity for the membrane and thus, it will be well taken up by this membrane (Dias et al., 2007, Mohammed et al., 2014). Therefore, the solubility parameter, which is defined as the sum of the cohesive forces of the molecule, is one of the values taken into account when choosing the vehicle (Hansen, 2007). Finally, hydrophilic solvents with solubility parameter values (δ) close to those of the active, are expected to promote permeation by shifting the δ values of the skin towards the δ value of the EA (15.4 (cal/cm^3^)^1/2^), thereby increasing the solubility and partitioning of the active inside the membrane (Lane, 2013, Liron and Cohen, 1984).

### 3.5 Permeation studies

**Fig. 6:**
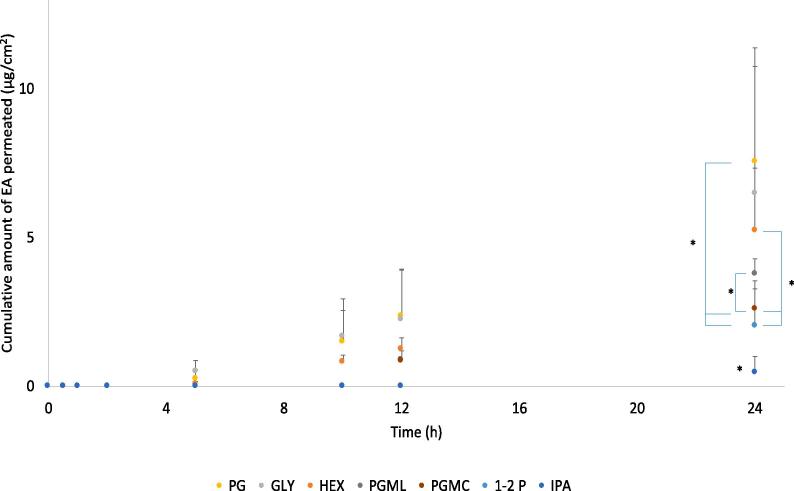
Cumulative permeation of 3-O-ethyl-l-ascorbic acid over time for single solvent systems (n ≥ 5, mean ± SD, *p < 0.05).

Finite dose permeation studies of EA were performed using GLY, PG, HEX, 1-2P, 1-5P, IPA, DiPG, TriPG, TC, PGMC and PGML. All permeation studies were conducted for 24 h with samples (200 μl) taken at different time points (t = 0, 15 min, 30 min, 1 h, 2 h, 5 h, 8 h, 10 h, 12 h and 24 h). A dose of 5 μl/cm^2^ of 2% w/w EA formulation was applied on the surface of the skin. The selection of solvents covered a range of solubility parameter values from 17.1 to 9.4 (cal/cm^3^)^1/2^, with GLY being the most hydrophilic and PGML the most hydrophobic as previously shown in Fig. 3. Permeation of EA was evident for GLY, PG, 1-2P, HEX, IPA, PGMC and PGML, as shown in Fig. 6. The highest amount of EA that permeated at 24 h was from PG (7.54 ± 3.22 μg/cm^2^), followed by GLY (6.49 ± 4.89 μg/cm^2^) and HEX (5.23 ± 2.11 μg/cm^2^); however, the values were not significantly different from each other (p > 0.05).

PG, 1-2P and HEX are hydrophilic low molecular weight 1,2 glycols that have been used in personal care products as solvents, skin and hair conditioning agents and viscosity increasing agents (Johnson et al., 2012). With regards to HEX, the ability of this glycol to promote skin permeation has not been investigated in human skin to date. This is the first study to assess the permeation enhancing properties of HEX using pig skin.

PG has been used for many years as a penetration enhancer for a number of compounds, however the mechanism of action has not been elucidated. It is considered that PG may act by increasing the partition or solubility of a drug in the *stratum corneum* (Lane, 2013). Pudney et al., 2007, Mohammed et al., 2014 used Confocal Raman spectroscopy to monitor the fate of vehicles and the actives as they permeate in humans *in vivo*. They found that the penetration of *trans*-retinol and niacinamide respectively was highly correlated with the depth of penetration of the PG. Brinkmann and Müller-Goymann (2005) used DSC, WAXD and SAXD to examine the effects of PG on human *stratum corneum*. They proposed that the molecule integrates into hydrophilic regions of hexagonally ordered lipids, thereby increasing the distance of the lipids and resulting in higher flux.

The exact mechanism of action of 1-2P as a skin penetration enhancer is not clear, it has been shown to promote increased delivery of a number of compounds. Duracher et al. (2009) found that 1-2P enhanced the permeation of caffeine in porcine skin and Heuschkel et al. (2008) showed that mixtures of 1-2P with 1–2 butanediol resulted in enhanced permeation of dihydrovenavenanthramide D across full thickness human skin. Recently, Steiner and co-workers (2017) examined the fate of 1-2P when applied to pig skin; 1-2P evaporated rapidly and only a quarter of the initial dose could be recovered after 24 h. Therefore, 1-2P can increase the thermodynamic activity of the active over time. In the present work single solvent systems of 1-2P did result in permeation of EA (2.01 ± 1.27 μg/cm^2^). However, as shown in Fig. 6, both PG and HEX delivered significantly higher cumulative amounts of EA than 1-2P (p < 0.05).

The solvent GLY is used in cosmetics as a humectant and skin-conditioning agent. The effect of GLY on the *stratum corneum* hydration has been proposed to result in a penetration enhancing effect. Bettinger et al. (1998) reported enhanced permeation of hexyl nicotinate acid *in vivo* in glycerol-treated sites of human subjects. This phenomenon was attributed to the interactions of glycerol with the *stratum corneum* lipids, enhanced desmosomal degradation, and the hydrating effect of glycerol. Brinkmann and Müller-Goymann (2005) investigated the effects of various solvents on human *stratum corneum* by DSC, WAXD and SAXD and they proposed that GLY intercalates between the hydrophilic heads of the orthorhombically packed lipids of the *stratum corneum*. This resulted in an increase of the gaps in the lipid bilayers, altering the *stratum corneum* barrier in a similar manner as PG.

Regarding the influence of the lipophilic solvents, permeation of EA for PGML (3.76 ± 0.54 μg/cm^2^) was significantly higher than PGMC (2.59 ± 0.96 μg/cm^2^, p < 0.05). The ability of PGML to act as a skin penetration enhancer has been reported previously (Haque et al., 2017, Hirata et al., 2013, Mohammed et al., 2014). Parisi et al. (2016) suggested that the presence of unesterified lauric acid in PGML may contribute to the enhancing effect of the solvent on the permeation of the active. All solvents delivered significantly higher amounts of EA comparing to IPA (0.45 ± 0.55 μg/cm^2^, p < 0.01). For the solvents PG, GLY, HEX, permeation of EA was detected after 5 h; however, permeation of EA for 1-2P, PGML, PGMC and IPA was not detected until 12 h (Fig. 6).

The results for mass balance studies and percentage permeation of applied EA are shown in Table 2. In general, the percentage permeation values of EA for the solvents follow the same order as the cumulative amounts permeated. The percentage permeations for the PG, HEX and GLY were similar, being 7.14, 6.06 and 5.39% respectively. However, the percentage EA permeation for 1-2P (2.01%) was significantly lower than PG and HEX (p < 0.05). For the hydrophobic solvents, the percentage EA permeation at 24 h for PGML (5.58%) is significantly higher than PGMC (3.93%, p < 0.05). All solvents resulted in higher percentage permeation comparing to IPA (0.60%, p < 0.05). This may reflect the rapid evaporation of IPA compared to the other vehicles.

**Table 2:** Cumulative permeation and percentage (%) permeation, skin extraction, recovery from skin surface and total recovery of EA for neat solvents (mean ± SD, n ≥ 5).

Solvent	Cumulative permeation (µg/cm^2^)	Percentage (%) of applied dose			
		% extraction	% permeation	% washed from the surface	% total recovery
GLY	6.49 ± 4.89	8.84 ± 6.74	5.39 ± 4.04	52.78 ± 29.18	69.34 ± 18.94
PG	7.54 ± 3.22	19.45 ± 5.98	7.47 ± 3.05	42.07 ± 14.01	72.92 ± 16.65
1-2P	2.01 ± 1.27	10.18 ± 2.90	2.01 ± 1.29	82.15 ± 11.03	94.34 ± 7.94
HEX	5.23 ± 2.11	18.55 ± 3.09	6.07 ± 2.36	25.25 ± 10.47	56.51 ± 10.78
TC	0.00 ± 0.00	12.01 ± 8.46	0.00 ± 0.00	45.92 ± 7.58	65.16 ± 6.27
IPA	0.46 ± 0.55	20.30 ± 6.57	0.60 ± 0.71	40.89 ± 9.05	61.79 ± 10.50
1-5P	0.00 ± 0.00	0.21 ± 0.41	0.00 ± 0.00	85.72 ± 32.47	87.72 ± 29.57
PGML	3.76 ± 0.54	15.93 ± 2.69	5.58 ± 0.79	51.89 ± 13.30	73.41 ± 11.99
PGMC	2.59 ± 0.96	20.94 ± 4.25	3.93 ± 1.42	74.68 ± 10.29	99.55 ± 8.64
DiPG	0.00 ± 0.00	13.92 ± 17.03	0.00 ± 0.00	72.41 ± 14.79	86.79 ± 13.21
TriPG	0.00 ± 0.00	0.70 ± 0.67	0.00 ± 0.00	97.43 ± 5. 73	101.87 ± 5.25

The percentage skin retention of applied EA was similar for HEX, PG, IPA and PGMC, ranging from 18.55 to 20.94% (p > 0.05). Compared with these solvents, the percentage retention of EA was significantly lower for GLY (8.84%, p < 0.05). The solvents HEX, PG, IPA and PGMC resulted in higher percentage retention of EA compared with 1-2P (10.18%, p < 0.05). The solvents TC, DiPG and PGML deposited comparable percentages of EA, namely 12.01, 13.92 and 15.93% respectively and these values were not different than HEX, PG, IPA and PGMC (p > 0.05). TC and IPA are hydrophilic solvents that both have previously been reported to increase the permeation and retention of various drugs across the skin (Brinkmann and Müller-Goymann, 2005, Chadha et al., 2011, Harrison et al., 1996, Oliveira et al., 2012). Haque et al. (2017) showed that skin penetration of anthramycin correlated with permeation of the vehicles. TC permeated rapidly through human skin, resulting in a plateau of drug permeation in the absence of the solvent. Considering the high retention of EA inside the skin together with the negligible permeation for either TC or IPA, it is likely that the rapid evaporation of the solvents may have resulted, similarly, in deposition of EA in the skin. A significantly higher percentage of EA retention was observed for PGMC (20.94%) compared with PGML (15.93%, p < 0.05).

1-5P has been reported to have antimicrobial and antifungal properties in pharmaceutical applications and it can be used as a moisturizing substance and solvent. Penetration enhancement tests *in vitro* showed 1-5P to be a penetration enhancer for certain pharmaceutical drugs in human skin, such as terbinafine (Evenbratt and Faergemann, 2009) and triiodothyroacetic acid (Faergemann et al., 2005). However, Møllgaard and Hoelgaard (1983) proposed that 1-5P has no effect on the barrier function and showed that compared with other diols, 1-5P delivered the lowest amount of estradiol across human skin. Here, we found no penetration of EA when we used neat 1-5P as solvent (Table 2). The solvents DiPG and TriPG are hydrophilic oligomers of PG with low molecular weights that are used as solvents, viscosity decreasing agents, and humectants (Fiume et al., 2012). Haque et al. (2017) showed that the percentage permeation of DiPG in human skin after finite dosing was 2.6% at 24 h. Fasano et al. (2011) used human skin to validate a mathematical model for estimation of permeation and predicted that the amount of TriPG penetrating the skin is expected to be 2-fold lower than DiPG. The low amounts of penetration observed here may reflect the limited permeation of these solvents through the skin. Haque et al. (2017) showed that when DiPG was used as a vehicle for anthramycin, it resulted in no permeation of the drug across the skin membrane. The total recovery after mass balance ideally should be 100 ± 15% (SCCS, 2010). The total recovery of the active for PG, GLY, HEX, PGML and 1-2P was 74.86%, 67.05%, 56.51%, 77.98% and 97.74% respectively. Total recovery of EA was lower than the recommended values, reflecting partial degradation of EA inside the skin. Studies are ongoing with human skin equivalent tissues in order to elucidate the breakdown of EA.

## 4 Conclusions

EA has been used in cosmetic products as a stable alternative to AA for a number of years. Additionally, EA containing products have been used for skin whitening and topical EA has been reported to be effective in treating medical conditions of hyper-pigmentation, such as melasma. Finally, because of its reducing ability, EA should be investigated as an antioxidant ingredient to increase stability of pharmaceutical formulations. To our knowledge this is the first report that characterizes the physicochemical properties of the molecule. This study is the first to report *in vitro* permeation and skin uptake data from a number of neat solvents under finite dosing. Among the hydrophilic solvents, PG, GLY and HEX delivered higher amounts of EA through the skin compared with the other vehicles, while for the lipophilic solvents, PGML resulted in significantly higher cumulative permeation of EA than PGMC. Knowledge of the physicochemical properties of EA, as well as the effect of single solvents will enable a selection of the most appropriate solvents for future formulation development of EA as well as of other molecules with similar physicochemical properties. Development of binary, ternary and more complex solvent systems that synergistically enhance penetration of the molecule will be the subject of a future publication. The stability of EA warrants further investigation, given some of the low recovery values observed. The identification and substantial quantification of the EA degradants should allow a greater understanding of the fate of EA inside the skin and will be also be the subject of future work.
