# Supplementary material for: 3-O-ethyl-l-ascorbic acid: Characterisation and investigation of single solvent systems for delivery to the skin
Source: Int J Pharm X. 2019 Jul 19;1:100025. doi: 10.1016/j.ijpx.2019.100025 (PMC6733298; doi:10.1016/j.ijpx.2019.100025)
Supplement: Supplementary data 1 [file mmc1.pdf]

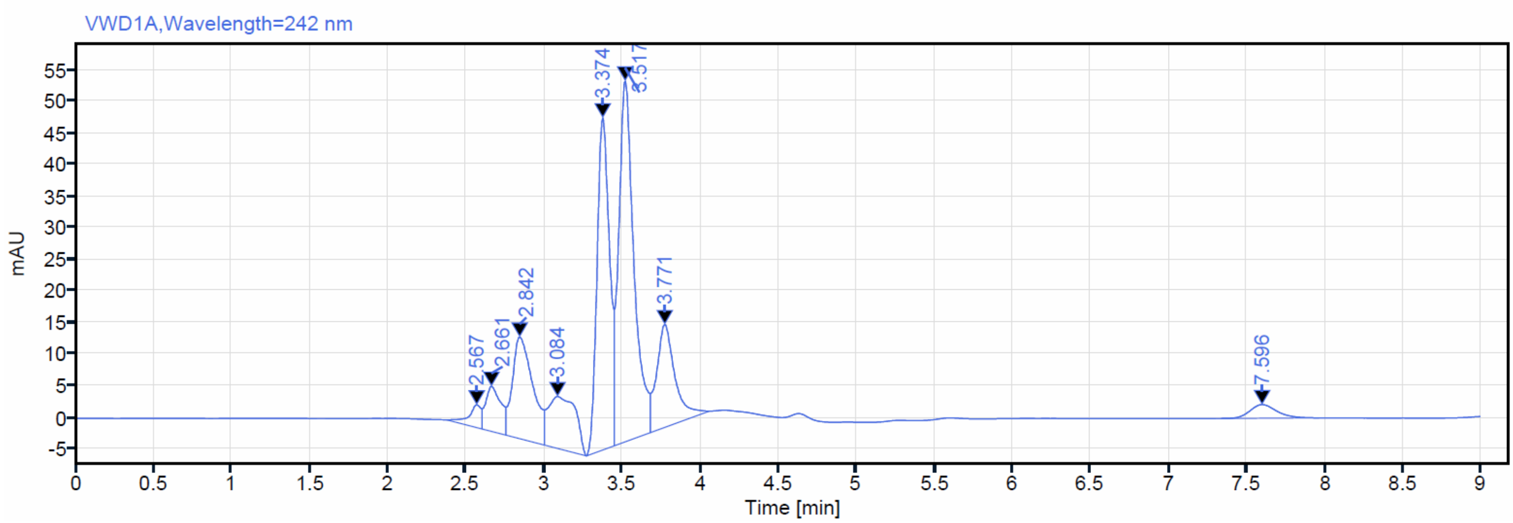

Figure S1 – Sample HPLC chromatogram of EA detected in the receptor solution during permeation study at the 12h time point.

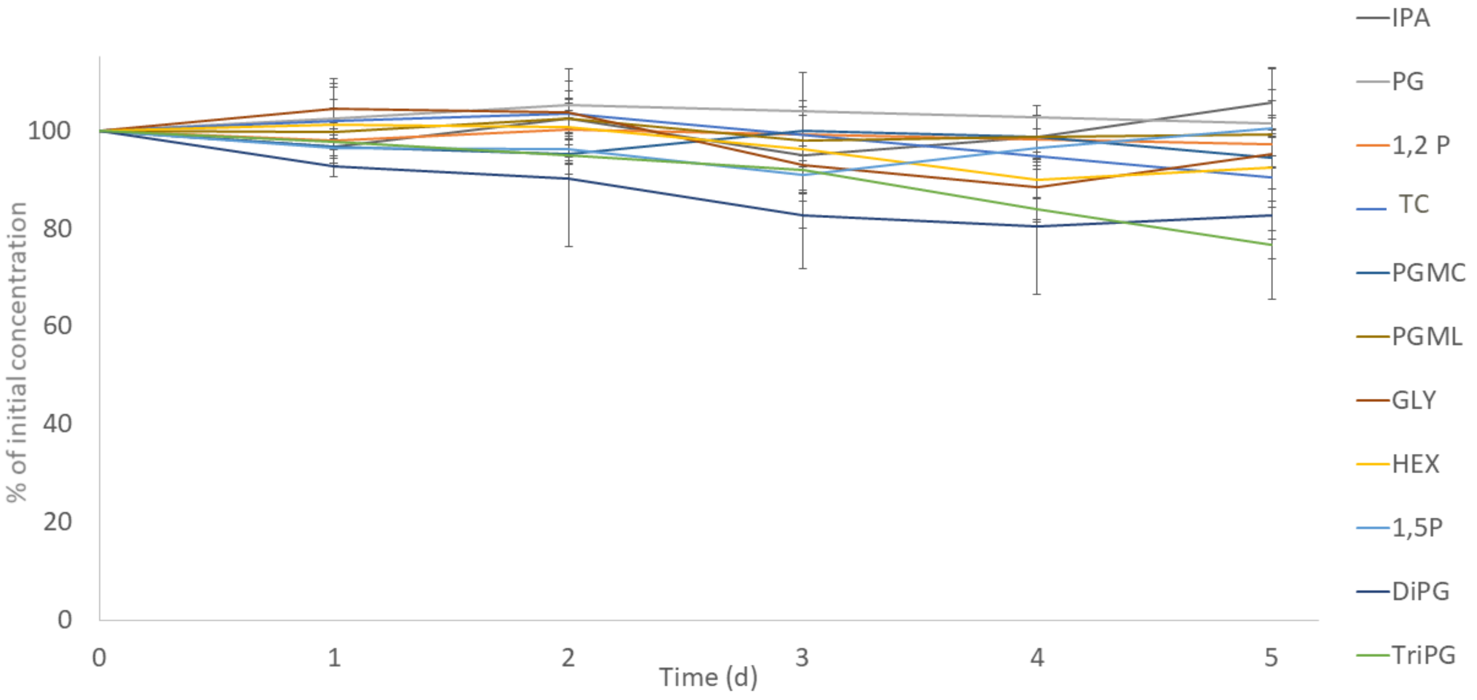

Figure S2 – Stability of 2 % (w/w) EA in various solvents over a 5-day period (mean ± SD, n=3).
